# Cover crop influence on pore size distribution and biopore dynamics: Enumerating root and soil faunal effects

**DOI:** 10.3389/fpls.2022.928569

**Published:** 2022-08-30

**Authors:** Maik Lucas, Linh T. T. Nguyen, Andrey Guber, Alexandra N. Kravchenko

**Affiliations:** ^1^Department of Soil System Science, Helmholtz Centre for Environmental Research – UFZ, Halle, Germany; ^2^Department of Plant, Soil and Microbial Sciences, Michigan State University, East Lansing, MI, United States

**Keywords:** X-ray CT, cover crops, soil structure, macropores, biopores, pore structure

## Abstract

Pore structure is a key determinant of soil functioning, and both root growth and activity of soil fauna are modified by and interact with pore structure in multiple ways. Cover cropping is a rapidly growing popular strategy for improving agricultural sustainability, including improvements in pore structure. However, since cover crop species encompass a variety of contrasting root architectures, they can have disparate effects on formation of soil pores and their characteristics, thus on the pore structure formation. Moreover, utilization of the existing pore systems and its modification by new root growth, in conjunction with soil fauna activity, can also vary by cover crop species, affecting the dynamics of biopores (creation and demolition). The objectives of this study were (i) to quantify the influence of 5 cover crop species on formation and size distribution of soil macropores (>36 μm Ø); (ii) to explore the changes in the originally developed pore architecture after an additional season of cover crop growth; and (iii) to assess the relative contributions of plant roots and soil fauna to fate and modifications of biopores. Intact soil cores were taken from 5 to 10 cm depth after one season of cover crop growth, followed by X-ray computed micro-tomography (CT) characterization, and then, the cores were reburied for a second root growing period of cover crops to explore subsequent changes in pore characteristics with the second CT scanning.

Our data suggest that interactions of soil fauna and roots with pore structure changed over time. While in the first season, large biopores were created at the expense of small pores, in the second year these biopores were reused or destroyed by the creation of new ones through earthworm activities and large root growth. In addition, the creation of large biopores (>0.5 mm) increased total macroporosity. During the second root growing period, these large sized macropores, however, are reduced in size again through the action of soil fauna smaller than earthworms, suggesting a highly dynamic equilibrium. Different effects of cover crops on pore structure mainly arise from their differences in root volume, mean diameter as well as their reuse of existing macropores.

## Introduction

Soil structure, i.e., the arrangement of solids and pores, defines most soil functions and processes (Rabot et al., 2018). Plant roots are the main modifiers of the pore structure, affecting it through a variety of mechanisms, including direct creation/modification of soil pores, increases in soil organic matter (SOM), exudation of mucilage, and water uptake (Gregory, 2022). A growing root interacts with pore structure in several ways: (1) roots can elongate into the soil matrix containing only the pores smaller than the root diameter (2) they can grow along an existing pores including biopores and (3) they can negotiate an existing pore at some angle before penetrating the soil (Jin et al., 2017; Lucas, 2022). During their growth, roots overcome soils penetration resistance and compress exiting pores creating new biopores (Dexter, 1987; Lucas et al., 2019a). Upon the root’s death and decomposition the biopores created by it can be reused by the subsequently growing plants. White and Kirkegaard (2010) showed that in 0.3–0.6 m depth 32–47% of wheat roots were located in biopores and below 1 m all of the roots were found in them.

Cover cropping is a promising technique for enhancing agricultural sustainability, known to increase SOM (Syswerda et al., 2011; Blanco-Canqui et al., 2015), improve pore structure, and benefit soil hydraulic properties including hydraulic conductivity (Haruna et al., 2020; Ogilvie et al., 2021). Among the benefits of cover crops is that they increase macroporosity and pore connectivity, create biopores, which can be reused by the main crops, thus positively influencing yields and root densities of the following main crop, especially in dry summers (Williams and Weil, 2004; Chen and Weil, 2010). It should be noted that the duration of the active cover crop growth in the US Midwest agriculture hardly exceeds 2–3 months, following the main crop and prior to growth termination in winter. Yet, even that time appears to be sufficient for generating pore structure benefits reported in cover crop studies (Haruna et al., 2020). Understanding the extent and mechanisms of soil pore formation during the short season of cover crop active growth and appreciation of its subsequent influence on pore structure is needed for making informed decisions on cover crop management and use.

Cover crop species typically used have a variety of contrasting root architectures, which comes along with potentially different effects on pore structure (Bodner et al., 2014; Cercioglu et al., 2018; Bacq-Labreuil et al., 2019). For example, in a recent review, Lu et al. (2020) examined the effect of root-induced changes of soil hydraulic properties and showed that coarse root systems increase macroporosity at the expense of smaller pores. The overall effect of roots, however, depended on total root volume (Lu et al., 2020). Haruna et al., 2020 reviewed the effect of cover crops on bulk density. Their results indicate that cover crops increase macroporosity by approximately 33% and total porosity by 4% especially in high clayey soils. However, many studies showed no effect of cover crops which may be caused by a short time of cover crop usage (Haruna et al., 2020). In an extensive study Bodner et al. (2014) investigated the effect of 12 different cover crops including *Phacelia tanacetifolia, Raphanus sativus* and *Vicia sativa* on pore size distribution in a silty loam soil. Their results showed exponential positive relationship between root volume density and total porosity. In addition, the authors showed that plant species with coarse root systems, and high median root radius increased macroporosity by more than 30% and decreased volume? of pores <15 μm Ø diameter, while fine root systems induced heterogenization of the pore space by increasing the volume pores <15 μm. The authors assumed that the first rooting types mainly create new growth paths, while the later root type with high root length densities and low penetration strength use mainly existing growth paths (Bodner et al., 2014).

Once the pores are modified by the initial impact of cover crop root systems, the newly-formed pore architecture is being further altered by roots of the subsequently grown plants, and by the resident soil fauna. Soil macrofauna (e.g., earthworms, termites, ants) and mesofauna (e.g., enchytraeids) move soil particles and create pores consistent with their sizes (van Vliet et al., 1998; Yunusa and Newton, 2003; Coleman et al., 2004). Especially earthworms create large biopores and also modify the biopore walls by secretion and compacting the surroundings (Kautz, 2015). What remains unknown is how substantial can be the influence of new plant growth on the cover crop-formed pore characteristics, and what contribution to the changes in the pore systems is made by the soil macrofauna.

The goal of this study is to quantify the influence of cover crops with contrasting root types on pore formation in a freshly tilled soil and their subsequent effect on the initially formed structure during a 2nd plant growing period of the same cover crops. Our objective is to investigate the effect of plant roots and soil fauna on pore size distribution, which presumably will be larger for roots with large root diameters and in freshly tilled soil in contrast to a second season with an existing biopore system. As on the field scale the exclusion of plants and soil fauna, which is needed for a real control, hardly can be achieved, we investigated all biological process leading to changes in pore structure using X-ray CT. For this we described the changes in the pore structure for the two root growing periods and linked them to the root growth paths through the soil, the dynamics in biopores (destruction and renewal); in addition we estimated the agents, e.g., root vs. faunal activities, which destroyed biopores.

## Materials and methods

### Study area and sampling

The samples for this study were taken from five cover crops grown in a randomized complete block design experiment located at Kellogg’s Biological Station (KBS), Michigan (42°24′07′′N 85°22′32′′W). The soil on the experimental site is Alfisol with a sandy loam texture. The studied cover crops are Annual Ryegrass (AR, *Lolium multiflorum*), Saber Oat (OA, *Avena sativa*), Dwarf Essex Rapeseed (DER, *Brassica napus*), Oilseed Radish (OR, *R. sativus*) and Austrian Winter Pea (AWP, *Pisum sativum*). AR is characterized by a highly branched and dense fibrous root system, OA has a branched fibrous root system, and DER, OR, and AWP develop tap roots of different sizes (OR > DER> > AWP) and extensive branching of increasing order roots (OR ~ DER < AWP). The cover crop trial experiment consisted of 1.3 m x 4.6 m plots, with 3 replicated plots per cover crop. Prior to cover crop planting in 2019, the experimental site was planted with oats. After oats was harvested for haylage in August of 2019 the field was conventionally tilled with a chisel plow (20 cm depth) followed by a field cultivator, and then planted to cover crops. In October 2019 two undisturbed soil cores (5 cm Ø) were taken from 5 to 10 cm depth from each of the three replicated plots (*n* = 6 per cover crop species). The cores were stored at 4°C and subjected to X-ray CT shortly upon collection.

In August 2021, immediately after the cover crop planting, the previously CT-scanned cores were buried into the replicated plots of the same cover crop species from which they originated. Note that in 2021, the cover crop trial experimental site was adjacent to but not at exactly the same location as that in 2019. Prior to being placed in the soil, each core had a polypropylene tube with perforations of 4 mm Ø (41% open area) stretched around it, hot glued and closed by caps with the same sized perforations and a centered opening of 2*2 cm (Supplementary Figure 1A). The 4 mm Ø openings enabled access by small and medium sized roots into the core, while the 2*2 cm opening in the cap allowed large taps roots to grow through the sample. The cores were placed back into the soil at 5–10 cm depth right next to the line of cover crop seeding. The cores were excavated in October 2021, after 69 days of cover crop growth, and subjected to the second X-ray CT scan. Upon excavation, it was apparent that many roots and earthworms were able to grow into the cores (Supplementary Figure 1B).

The aboveground cover crop biomass from each plot was obtained at the time of core excavation. For that a 0.25 m^2^ rectangle frames were randomly placed within the plots and aboveground biomass within the rectangle was cut and then dried at 60°C prior for dry weight determination.

### X-ray CT scanning

Soil cores were scanned using a X-ray microtomograph (X3000, North Star Imaging, Rogers, United States) with the same energy settings in 2019 and 2021 (75 kV and 450 μA). These settings led to a focal spot of 33.75 μm on the VarianL07 detector panel (size of 1920*1536 pixels). However, a continuous SubpiX mode was used to gain a resolution of 18 μm. During one scan, 2,880 projections were taken at 12.5 fps using an average of 4 frames for each of four subimages (2 rows and 2 cols). Image reconstruction was performed using the NSI reconstruction software.

### Image processing

The images pairs from 2019 and 2021 were registered using the elastix software to detect changes within the images, e.g., to differentiate new from old roots (Klein et al., 2010; Shamonin, 2013). The registration protocol was similar to the one used by Lucas et al. (2020) and implemented a multi metric registration combining Euclidean distance between the corresponding landmark points and the mutual information criterion (Mattes et al., 2001). The registered images were cut into cubes of 1850 × 1850 pixels with a height ranging between 2,100 and 2,300 pixels in Fiji (V. 1.53n; Ollion et al., 2013). This was done to reduce artifacts at the column wall. After this, a contrast enhancement (saturation value = 0.35) was performed, and the bit depth was reduced to 8-bit. Ring-artifacts were reduced using the wavelet-FFT stripe filter implemented in the Xlib plugin (Münch et al., 2009). A non-local means filter was applied (Darbon et al., 2008; Buades et al., 2011) using scikit-image (van der Walt et al., 2014) in Python (van Rossum and Drake, 2009) in order to ensure a good automatic threshold detection for pores. The later was performed by computing the threshold value from 7 different threshold detection methods using SimpleITK (V. 2.0.2., Beare et al., 2018), namely Otsu, Kittler, Triangle, Huang, IsoData, Maximum Entropy, Li, Renyi Entropy, Yen and Moments and calculating the mean of them. For the average, outliers (>1 standard deviation) were removed. This assemblage allowed for a robust calculation of a pore threshold (Schlüter et al., 2014).

Roots and biopores were segmented according to the Rootine script (Gao et al., 2019; Phalempin et al., 2021a) for roots and Lucas et al. (2019b) for biopores, respectively. Both scripts rely on the Tubeness plugin in Fiji to separate different sized tubular objects from the remaining irregularly shaped pore network. For this study, the two scripts were adapted to (1) allow segmentation in the subsamples without a column wall, (2) equalize important script parts to segment biopores and roots equally over their whole size range, (3) get the true biopore form and (4) increase the speed of the segmentation. The whole workflow was rewritten in Jython script language to use the multithreaded ImageJ Ops version of the tubeness filter (Rueden et al., 2021) and can be found on https://github.com/Maik-Lu/Roots_and_Biopores and the general workflow on Supplementary Figure 2. Summarized, the process consisted of the following steps: Binary X-ray CT images of the soil cores were used for biopore and root segmentation. To segment roots, a binary image was created with its foreground class containing water, roots, as well as other particulate organics (organic binary in Supplementary Figure 2). The upper and lower thresholds for this class were calculated using the threshold for pores (threshold/2.4 < gray value < threshold). The fraction of 2.4 turned out to be a robust value to describe the range of gray values from highest (threshold) to lowest gray value of roots and other organic material in the image dataset. This value may differ in image sets with a different contrast and therefore needs to be corrected manually by the user. The ground base for the biopore segmentation was binarized using the pore threshold only (pore binary in Supplementary Figure 2). These binaries were downscaled to 50 and 20% to apply Tubeness filters with σ -values between 1–4 and 2–30, respectively (step size =1). Misclassified objects were removed similar to Phalempin et al. (2021a) after combining all elongated objects. A Distance Transform Watershed 3D operation was performed (MorphoLibJ, Version 1.4.3; Legland et al., 2016) on the tubeness result to separate root laterals from roots higher order before filtering misclassified, i.e., blob-like objects. To separate new roots from old ones in the images from samples after the 2nd root growing period, the root images resulting from cores of the 1st root growing period were subtracted from the former ones. In the same way, biopores destroyed and newly created in the 2nd root growing period were computed by subtraction of the segmented images using difference images. The image subtracted was 3D dilated before subtracting to account for small changes in root/biopore thickness and position. Last, a size opening (<0.3 mm^3^) performed to get a clean image of roots/biopores. In one image an earthworm was falsely segmented as root and therefore removed manually.

The destroyed biopores were further classified into three groups based on the hypothesized agent of destruction, namely, those destroyed by earthworm casts, by mesofauna casts, or by unidentified causes (internal erosion or compaction by an unknown source). This classification was performed using a random forest classifier trained in ILASTIK (Berg et al., 2019) using the filtered gray value images masked by the binary image of the destroyed biopores. From each plant one image of a soil core was used to create the training data in which large earthworm casts were differentiated from smaller mesofauna casts, along with all other biopores that could not be assigned to either of the two. The out-of-bag error was <0.01. After this, a distance Transform Watershed 3D operation from MorphoLibJ (Version 1.4.3; Legland et al., 2016) was performed on the binary images to separate different biopore segments and to assign these segments to one of the three destruction agents. The later was based on the majority class in the corresponding segment.

### Image analysis

Pore size distributions (PSD), biopore size distributions and root size distributions were calculated on the binary image using the local thickness method in Fiji. This method is based on the maximum inscribed sphere method. We followed and quantified the trajectory of roots growing during the second cover crop season using the new root image as a mask on the segmented image from 2019, which contained the labels of pores, matrix, biopores and roots.

### Validating root segmentation by destructive sampling

The root segmentation performed using X-ray CT images was validated using traditional destructive root length analyses. After the second scan, the cores; were opened and the soil was washed through a 1 mm sieve to collect roots. The roots were stored in ethanol until scanned on a flatbed scanner (Epson Perfection V850 pro) at 1200 dpi. The analysis of root length was done using the Rhizovision Explorer (V. 2.0.3 Seethepalli et al., 2021).

### Statistics

Differences in pore, biopore, and root sizes among the studied cover crops after the first and the second season of growth, as well as their changes after the second season were assessed using linear mixed model approach implemented in the lme4-package (Bates et al., 2015) of R (V. 4.1.1). The statistical models included cover crops as the main studied fixed effect factor, while fixed effects of the season and pore size and their interactions were added as needed. The random effects of experimental plots nested within the cover crops and the intact soil cores nested within the cover crops and the plots were included in the models, with the former used as an error term for testing the cover crop effect. The assumptions of normality and homogeneity of variances were assess using normal probability plots of the residuals and Levene’s tests for equal variances, respectively. When the normality assumption was found to be violated, the data were log-transformed. When the equal variance assumption was violated, the unequal variance models were fitted using nlme-package in R. Multiple comparisons among the cover crop and season means within each pore size group were assessed using t-tests, conducted when respective F-tests were found to be statistically significant at *p* < 0.05 level. The results of such t-tests are presented using letter separations and no letters are shown in tables and figures when the respective F-tests were not statistically significant. The differences are reported as statistically significant at p < 0.05 and as trends at *p* < 0.1.

To find associations among different agents and pore size classes, we computed Pearson correlation coefficients using the R-package “lares” (V. 5.1.1). All significant (*p* > 0.01) correlations between pore size distribution and biopore and root size distribution data from the two seasons, as well as volumes of destroyed biopores and roots growing into the matrix are presented.

## Results

### Roots

Root length densities of the five studied cover crops determined non-destructively with X-ray CT were in a good agreement with destructively analyzed roots. A linear model with no intercept (crossing the origin) produced the regression slope of 0.99 for the relationship between the length densities measured destructively and those using X-ray CT (*p* < 0.001, Supplementary Figure 3). The *R*^2^ of 0.62 reflected variabilities and uncertainties involved in both methods. While X-ray CT may underestimate the roots of the smallest size classes, the destructive analysis can lead to losses of both small roots and large brittle ones, which likely separated into small pieces and were washed through the sieve.

The image pairs from two root growing periods, allowed to differentiate newly developed roots from roots from the previous main crop and cover crop. While DER and AWP developed comparably high root volumes, the lowest root volume densities were found in OA (Table 1). In addition, DER had the highest mean root diameter, which was, however, not significantly (*p* < 0.05) different from roots developed by other crops (Table 2).

**Table 1:** Distribution of pores, biopores and roots in cores of the five studied cover crop species.

Root growing period	Cover crop	Macroporosity (%)			Bioporosity (%)			Root density (%)			Plant biomass (g m^−2^)		
1st	AR^1^	16.70	±0.97	a	1.63	±0.28	a	0.23	±0.04	bc*	243.81	±21.08	a
2nd	AR	16.36	±1.19	a	1.84	±0.34	a	0.09	±0.03	ab*	356.79	±39.65	ab
1st	AWP^2^	18.50	±1.12	ab	2.15	±0.28	a	0.27	±0.06	c	287.21	±62.91	a
2nd	AWP	18.34	±0.81	a	2.23	±0.32	a	0.15	±0.03	b	346.17	±92.73	ab
1st	DER^3^	18.69	±1.20	ab	1.76	±0.32	a	0.31	±0.14	abc	298.76	±31.31	a*
2nd	DER	17.69	±1.43	a	2.30	±0.34	a	0.15	±0.04	b	613.40	±89.07	bc*
1st	OA^4^	20.88	±0.94	b	1.41	±0.36	a	0.11	±0.02	a*	414.10	±58.34	a
2nd	OA	18.84	±0.97	a	1.73	±0.29	a	0.05	±0.02	a*	230.49	±30.37	a
1st	OR^5^	20.38	±0.61	b	2.04	±0.29	a	0.14	±0.03	ab	444.03	±52.45	a*
2nd	OR	19.13	±0.76	a	2.19	±0.42	a	0.09	±0.02	ab	779.02	±191.32	c*

Shown are means and standard errors of the mean. Different letters indicate significant differences between the species within the root growing period (*p* < 0.05), while stars indicate significant differences between the root growing periods for a given cover crop. For the 1st root growing period only newly developed roots are shown, while results from the 2nd root growing period potentially contain roots from the previous main crop.

1 AR, Annual Ryegrass (*Lolium multiflorum*).

2 AWP, Austrian Winter Pea (*Pisum sativum*).

3 DER, Dwarf Essex Rapeseed (*Brassica napus*).

4 OA, Saber Oat (*Avena sativa*).

5 OR, Oilseed Radish (*Raphanus sativus*).

**Table 2:** Mean diameter of pores, biopores and roots (based on volumes) in cores of the five different plants.

Root growing period	Cover crop	Pore diameter (mm)			Biopore diameter (mm)			Root diameter (mm)		
1st	AR^1^	0.52	±0.07	a	1.27	±0.16	a	0.32	±0.02	a
2nd	AR	0.46	±0.07	a	1.21	±0.17	a	0.30	±0.02	a
1st	AWP^2^	0.69	±0.11	a	1.40	±0.19	a*	0.30	±0.02	a
2nd	AWP	0.61	±0.09	a	1.13	±0.16	a*	0.29	±0.02	a
1st	DER^3^	0.64	±0.09	a	1.23	±0.17	a	0.36	±0.05	a
2nd	DER	0.66	±0.10	a	1.37	±0.17	a	0.35	±0.06	a
1st	OA^4^	0.43	±0.07	a	1.14	±0.20	a	0.33	±0.07	a
2nd	OA	0.47	±0.07	a	1.37	±0.17	a	0.32	±0.06	a
1st	OR^5^	0.52	±0.07	a	1.39	±0.19	a	0.31	±0.05	a
2nd	OR	0.55	±0.09	a	1.50	±0.24	a	0.31	±0.04	a

Shown are means and standard errors of the mean. Different letters indicate significant differences between plants within the same root growing period (*p* < 0.05), while stars indicate significant differences between the root growing periods for a given cover crop. For the 1st root growing period only newly developed roots are shown, while results from the 2nd root growing period potentially contain roots from the previous main crop.

1 AR, Annual Ryegrass (*Lolium multiflorum*).

2 AWP, Austrian Winter Pea (*Pisum sativum*).

3 DER, Dwarf Essex Rapeseed (*Brassica napus*).

4 OA, Saber Oat (*Avena sativa*).

5 OR, Oilseed Radish (*Raphanus sativus*).

**Figure 1:**
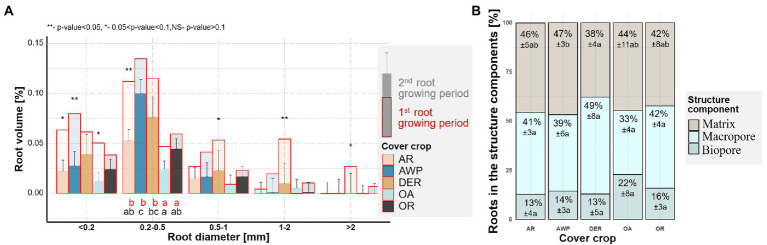
**(A)** Root size distribution for root volumes of the five studied cover crops after the 1st (red) and 2nd root growing period (multiple fill colors). Letters indicate significant differences between plant species within each size class for the 1st (red) and 2nd (black) growing period (*p* < 0.05). Stars above the bars show significant differences between the two growing periods. Whiskers show standard error of the means for the 2nd growing period. Stars above the bars show significant differences between the two growing periods. **(B)** Root growth into different structural components of the soil, namely, into existing biopores (>36 μm Ø, cylindrical shape), macropores (>36 μm Ø), and soil matrix (no pores visible at CT resolution) during the 2nd growing period. The numbers are means ±standard errors. Letters indicate significant differences between plant species within each structural component (*p* < 0.05).

The volumes and lengths of roots in <0.5 mm size classes (Figure 1A; Supplementary Figure 4) were larger after the 1st root growing period as compared to the 2nd one. These differences are due to the fact that after the 1st root growing period soil samples contained old not fully decomposed roots from the previous crop(s) that could not be reliably separated from those of the new cover crop growth. Thus, to assess the root growth of the studied cover crops, we will focus on the roots which grew into the soil cores during the 2nd root growing period.

In the second root growing period the highest volumes of newly grown cover crop roots were found in the size class between 0.2–0.5 mm Ø, in which AWP developed significantly larger amounts of roots compared to OA (Figure 1A). Largest differences between the cover crops species in root length densities, however, occurred in the smallest size class, with significantly (*p* value = 0.043) larger root length for DER compared to OA (Supplementary Figure 4). In the 0.5–1 mm root diameter class, DER had numerically the largest root volume compared to the other species.

More than half of the roots grew into biopores or macropores during the 2nd root growing period (Figure 1B), a trend especially pronounced in DER. Only 38% of DER roots grew into the soil matrix as compared to 47% of AWP roots. However, due to the lower root volumes of OA, significantly smaller total root volumes elongated into the dense soil matrix from OA compared to AWP (Supplementary Figure 5).

### Pores

After the 1st root growing period the total macroporosity was significantly higher in soil samples from OA and OR compared to AR (Table 1). The differences between the macroporosity created by the plants decreased during the 2nd root growing period. Although after the 1st root growing period AR samples had still the lowest macroporosity (16.4%) compared to AWP (18.3%), DER (17.7%), OA (18.9%), they were only significantly different from OR (19.1%).

**Figure 2:**
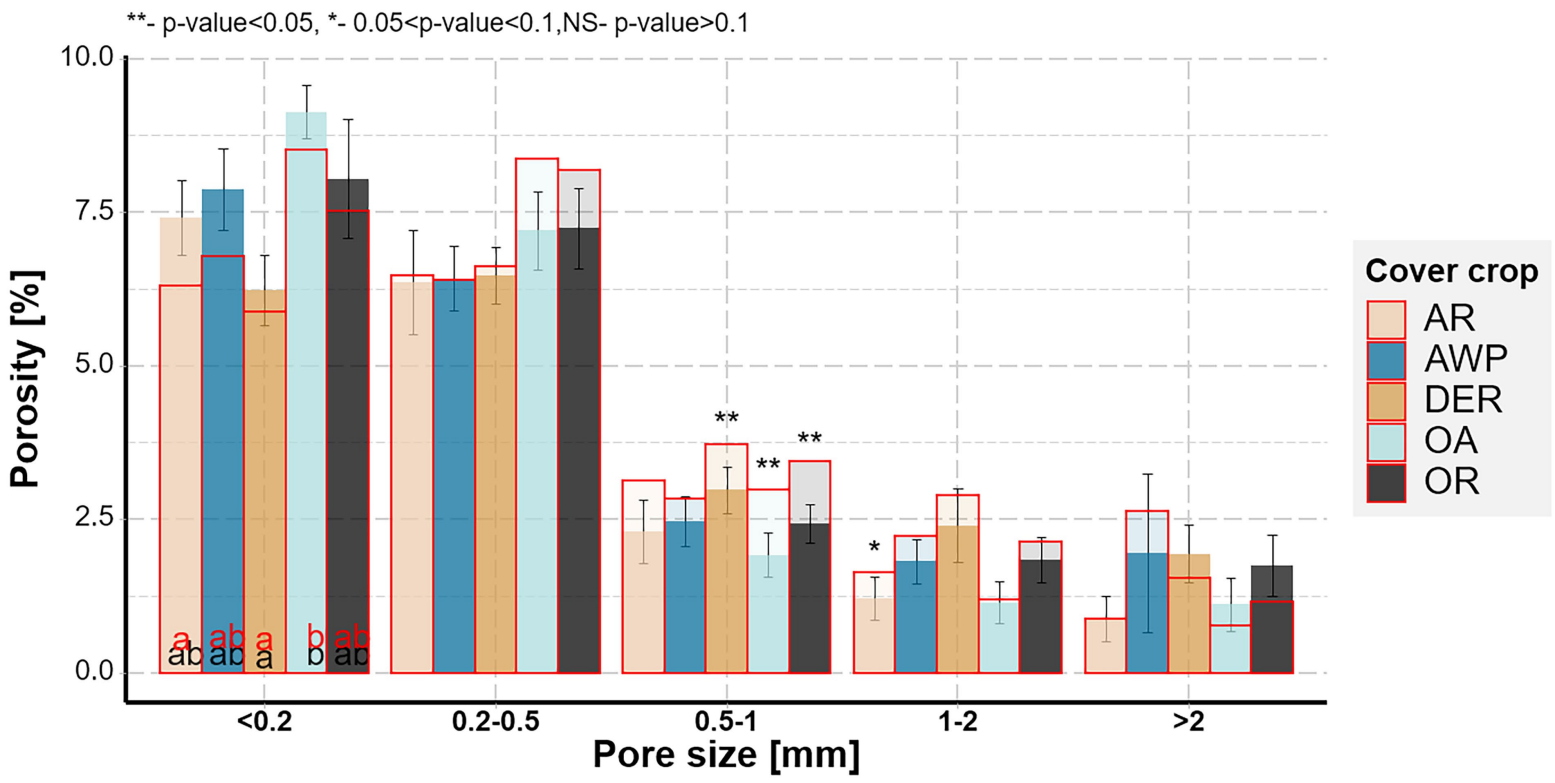
Soil pore size distributions for the five studied cover crops after the 1st (outlined in red) and the 2nd root growing period (multiple fill colors). Letters indicate significant differences between plant species within each size class for the 1st (red) and 2nd (black) growing period (*p* < 0.05). Stars above the bars show significant differences between the two growing periods. Whiskers show standard errors of the means after the 2nd growing period.

There was a significant time effect on the pore size distribution for pores in 0.2–0.5 mm Ø size range (Figure 2). Interestingly, there was a tendency for a reduction in volumes of larger pore size classes, while the smallest size class tended to increase during the 2nd root growing period for all plants. That indicated that most differences in pore size distributions among the cover crops systems occurred during the 1st root growing period. There was a significant plant effect on the smallest pores size class (<0.2 mm Ø), with OA having significantly higher pore densities compared to DER. Especially OA and AR had lower amounts of large pores (<1 mm Ø) compared to AWP, DER and OR.

### Biopores

**Figure 3:**
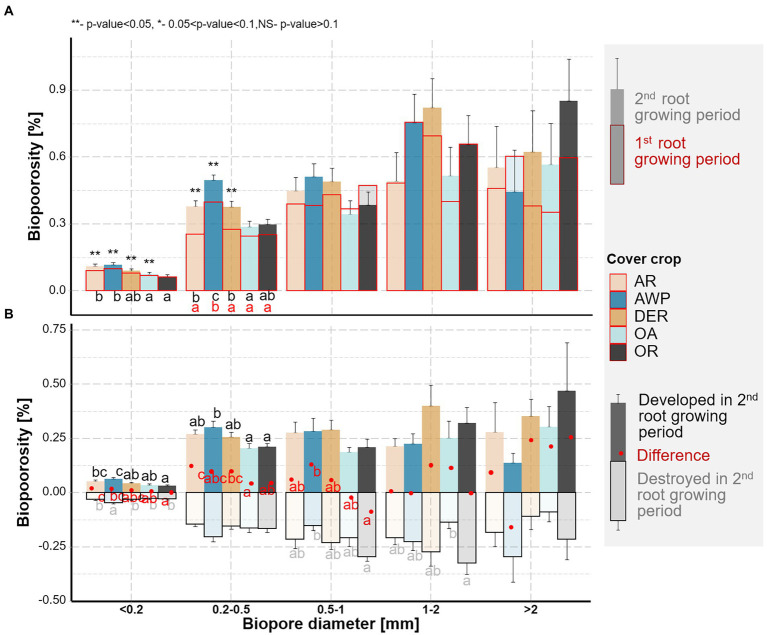
**(A)** Biopore size distribution of the soil samples from the five studied cover crops. Letters indicate significant differences among the cover crop species within each size class (*p* < 0.05). Biopores after the 1st root growing period are shown in red, those after the 2nd root growing period are shown in black. Stars above the bars mark significant differences between the two root growing periods. **(B)** Biopores destroyed (pale colors) and created (dark colors) during the 2nd growing period. Red points show the resulting total changes in bioporosity. Whiskers show standard errors of the means. Letters mark significant differences among the species within each size class for destroyed (pale), created (black), and total (red) biopores (*p* < 0.05).

Total bioporosity increased for all plants by the 2nd root growing period and was following a numeric trend DER > AWP > OR > AR > OA (Table 1). The biopore volumes in <0.2 mm and 0.2–0.5 mm size classes significantly differed among the plant species in both studied seasons and, overall, significantly decreased after the second season (Figure 3A). AWP developed the highest bioporosity in these two size classes. The largest volumes for biopores were found in the size classes 1–2 mm Ø and >2 mm Ø (Figure 3A).

In addition to estimating the total bioporosity in the samples, we were also able to compute the changes in the biopores that took place within the 2nd root growing period (Figure 3B). These data show large dynamics, especially for biopores <1 mm diameter, where more than half of the total volumes were destroyed and recreated. For biopores with 1–2 mm Ø and > 2 mm Ø, especially in OA, low amounts of biopores were destroyed, while for 1–2 mm sized biopores OR, and in the size class >2 mm Ø AWP higher volumes of destroyed biopores were observed over time. In contrast to the smaller size classes, in the diameter class >2 mm, AWP showed a reduction in bioporosity, which was, however, not significantly different compared to the other cover crops (*p* value >0.05).

We were further able to classify the bioporosity based on the agent leading to the destruction of the biopores. In AR, AWP, OR and OA the proportion of biopores filled by earthworm casts and by other mesofauna casts were similar. In DER, however, significantly greater proportion of biopores was blocked by other mesofauna excrements than by earthworm casts. Biopores, which were destroyed but could not be classified according to their cast (N.A.), accounted for the smallest fraction of the destroyed biopores, i.e., most of the biopores were destroyed through soil faunal activity. This class was most often completely filled through local compaction/particle shifts.

### Cover crop effects on porosity and pore size distribution

There was no effect of the mean root diameter on mean pore diameter (*p* value = 0.63). However, there was a significant increase in pore diameter (>0.036 mm Ø) with increasing biopore diameter (*p* < 0.01, *R*^2^ = 0.2) suggesting a change in PSD through the development of biopores.

**Figure 4:**
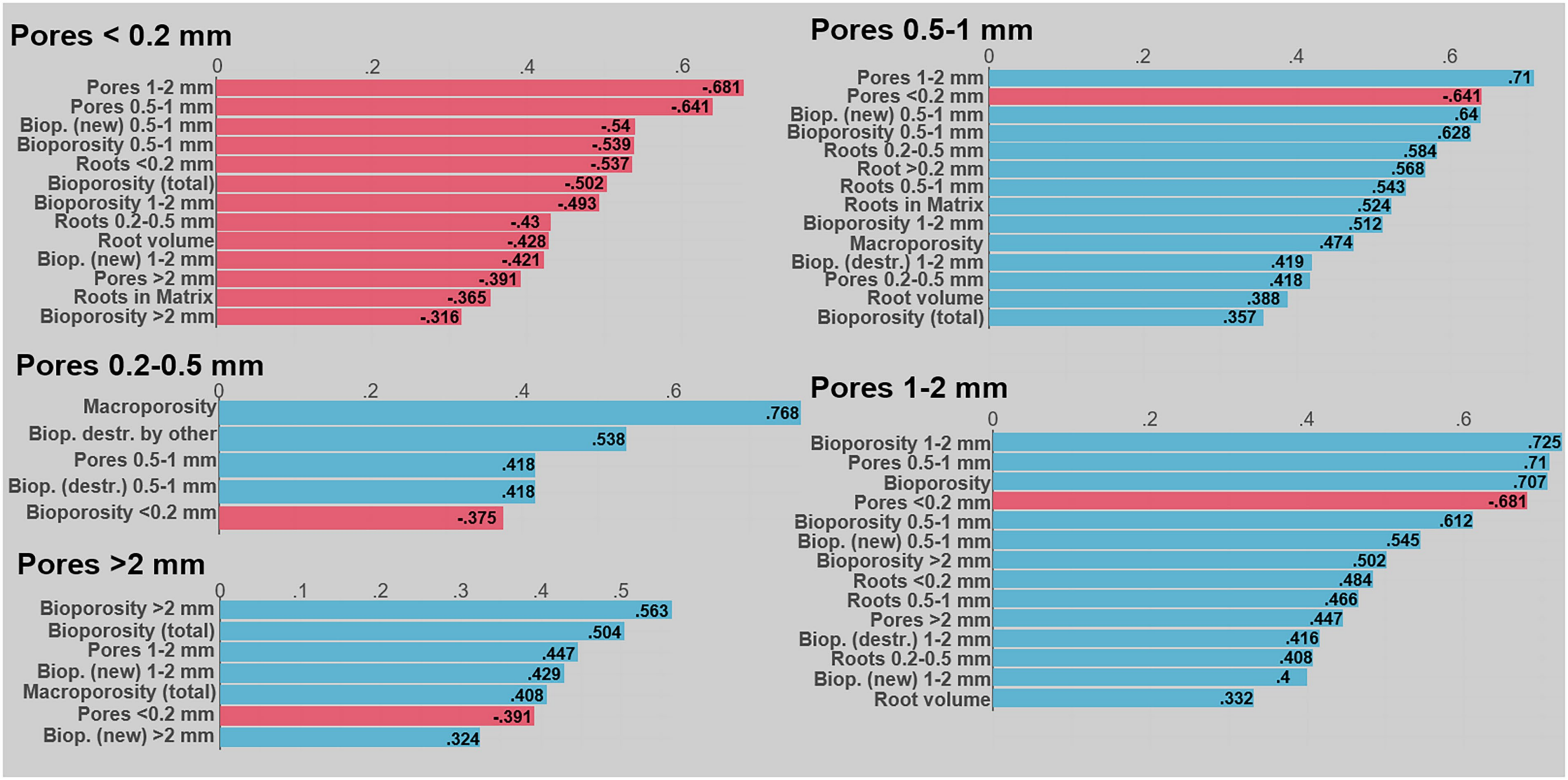
Associations between root, biopore, and pore characteristics and volumes of pores in different size classes visualized by Pearson correlation coefficients for the data from the 2nd root growing period. Shown are correlation coefficients significant at <0.1; red mark negative and blue positive correlations. The Biop. (destr) and Biop. (new) are the bioporosity destroyed and created during the 2nd growing period. Biop. (destr) by other are these biopores filled by cast other than earthworm cast (presumably from enchytraeids): Note that these analyses are performed using analyses of CT images after the 2nd root growth period and correlations therefore result from processes of both root growing periods.

Figure 4 shows associations between various root and biopore characteristics and volumes of pores of different size classes visualized by Pearson correlation coefficients. It is apparent, that the smallest pore size class is negatively correlated with all other measures, while all other pore size classes were positively correlated with many root and biopore size classes. This suggests, that the creation of biopores – partly by roots, partly by soil fauna – lead to an increase in larger macropores, while pores <0.2 mm Ø were compacted. Different root size classes and the total root volume as well as the volume of roots growing into the soil matrix was positively associated with macropore size classes of 0.5–2 mm Ø, while negatively related to macropores <0.2 mm Ø. However, we did not differentiate between biopores created by roots or ones created by the soil fauna in our image segmentation protocol. As the correlations of root size classes with different macropore size classes were always lower compared to the impact of biopores, i.e., the combined activity of roots and soil fauna, it can be suggested, that both agents influenced the pore system in a similar way during the two plant growing period. While both, roots and soil fauna created biopores of their size, they reduced macropores <0.2 mm Ø. Thus, while the cover crops species mainly accounted for increasing macroporosity between 0.5–1 mm Ø, the increase in pores >2 mm can be mainly attributed to the activity of earthworms creating large biopores. In addition, the destruction of biopores by mesofauna was positively associated with 0.2–1 mm Ø macropores during the second root growing period.

## Discussion

### General structure dynamics

We used X-ray CT scanning to study short-term pore structure formation in the intact cores from tilled topsoil under 5 different cover crops. In order to explore interactions of pore structure with plant roots and soil fauna, we followed the cores for a 2nd root growing period. It should be noted that, since we did not have control cores in the study (i.e., the cores that did not experience any influence of soil fauna and plant roots) the absolute effects of roots and fauna could not be estimated. However, the absence of such controls does not affect the assessments of the changes that took place during the 2nd root growing period as well as the comparisons among the studied plant species.

**Figure 5:**
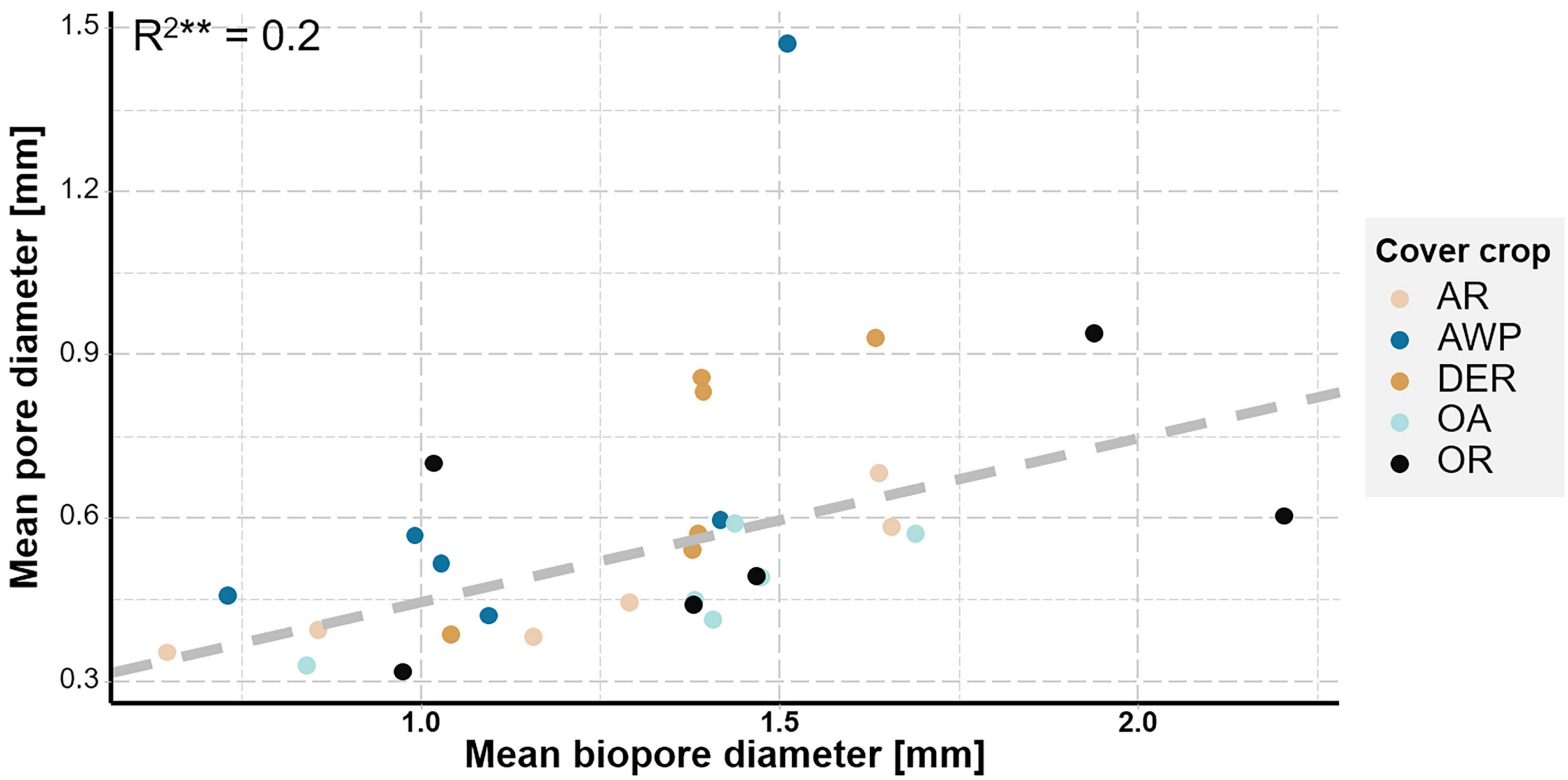
Mean pore diameter plotted versus mean biopore diameter. Colors represent the studied cover crop species. Dashed line represents the linear regression fitted to the data.

Our data clearly demonstrated, that the creation of biopores of all size classes by both roots and soil fauna reduced the volume of <0.2 mm Ø pores (Figure 4). The investigated plants developed relatively low quantities of thick roots >1 mm Ø (Figure 1), while large biopores (>1 mm Ø) constituted the biggest share of the observed bioporosity and were not affected by either time or plant species (Figure 3A). Therefore, it can be surmised that these biopores were not created solely by the studied cover crops. Stolze et al. (2022) demonstrated that root biomass directly correlates with small-sized biopores, while the density of anecic earthworms corresponded to larger ones. The effect of large biopores created by earthworms on total porosity, however, can vary substantially. Nevertheless, in compacted soils they were shown to reduce soil bulk density by increasing macroporosity (Ponder et al., 2000; Lang and Russell, 2020; Meurer et al., 2020). However, ploughing and seedbed preparation for cover crops leads to a destruction of most of the large biopores and the overall pore system gets disrupted and unconnected (Kautz, 2015; Lucas et al., 2019b). Roots and soil fauna exploring the soil after ploughing prior to the 1st root growing period, therefore had to create new pathways throughout the soil by producing biopores at the expense of smaller pores. This effect is also visible in the strong positive correlation between mean biopore diameter and mean pore diameter—the more biopores were created the greater became the overall pore size (Figure 5). Yet, total macroporosity was positively correlated with 0.2–1 mm and >2 mm Ø pores, but it was not negatively correlated with <0.2 mm Ø pores. Thus, large biopores were created partially through a shift in pore size distribution and partially through an increase in total porosity.

However, after a sufficient network of biopores has formed, they can be reused or rearranged, resulting in only minor changes in bioporosity and thus pore size distribution (Han et al., 2015a,b; Lucas et al., 2019b). This rearrangement was also visible in the 2nd root growing period of this study, during which a large number of biopores were destroyed while at the same time new biopores were formed. (Figure 3B). In addition, large amounts of macro- and biopores were reused by roots (Figure 1). Therefore, only small changes in (bio-)pore size distribution (Figures 2, 3A) were observed between the two root growing periods. Yet, biopores <0.2 and 0.2–0.5 mm Ø, which were most likely affected by the cover crop roots (Figure 1; Supplementary Figure 1) increased during the 2nd root growing period. Thus, <0.2 mm Ø pores showed a tendency for an increase over the 2nd root growing period and were not further reduced through the creation of large sized biopores as visible in the 1st root growing period (Figures 2, 4).

**Figure 6:**
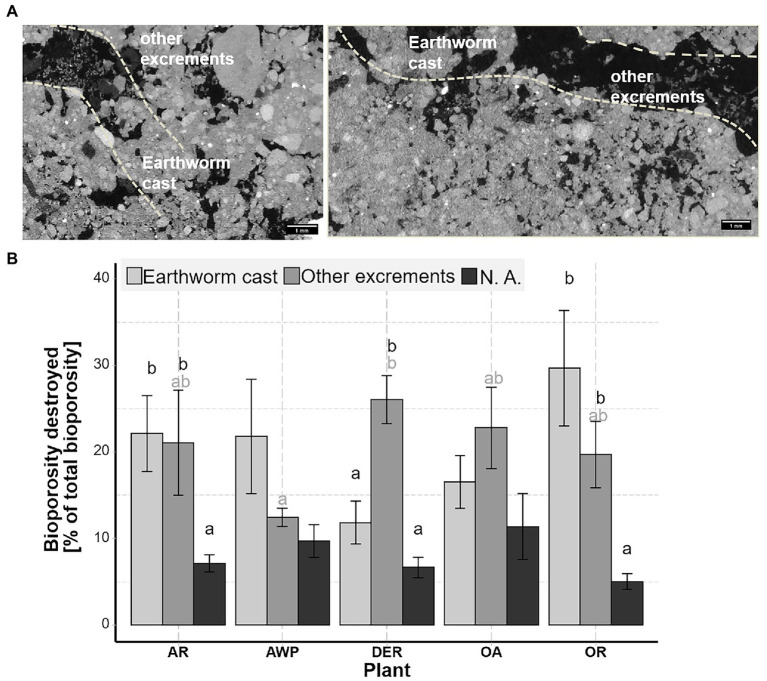
Destroyed bioporosity through earthworms, mesofauna and other causes (e.g., by internal erosion). **(A)** Shows X-ray CT image slices of biopores filled by earthworm and mesofauna cast. Distribution of destroyed biopores by different causes for the five different cover crops expressed as percent of the total bioporosity. **(B)** Whiskers show standard error of the mean. Black letters indicate significant differences between the three components for each plant, while significant plant effects are indicated by the gray colored letters (only significant for other excrements).

Although we did not analyze the activity of soil fauna directly, the segmentation of destroyed biopores with respect to their filling allow inferences regarding the activity of soil fauna and their reuse of existing biopores (Figure 6). Many biopores were filled by earthworm casts or casts of smaller soil fauna. The latter appeared to be largely excreta of enchytraeids, as suggested by analysis of thin sections in the literature (Davidson et al., 2002; Baveye et al., 2022) and may account for up to 30% of the area (Davidson et al., 2002). Enchytraeids were shown to increase pores of their size (0.050–0.2 mm) and their egestion of the soil also results in destabilization and filling of macropores, causing an increase in smaller pores (van Vliet et al., 1998; Coleman et al., 2004). Indeed, biopores blocked by smaller cast still contained a large amount of narrow macropores (Figure 6A) and their destruction therefore was positively correlated with pore sizes between 0.2–0.5 mm Ø (Figure 4). Similar to earthworms, enchytraeids ingest both organic and mineral particles, although typically of smaller size ranges and there is an evidence that enchytraeids consume larger fecal castings of earthworms (Coleman et al., 2004). Indeed, large amounts of excrements, which were highly organic (based on the image gray values), were found next to earthworm casts and old root debris (Figure 6A). Yet, it cannot be excluded, that some of these smaller particles were misclassified as excrements and result from internal erosion.

In summary, our results demonstrate that after ploughing (i.e., the 1st growing period of this study) biopores are created through earthworm and root activities at the expense of smaller pores. If the soil remains undisturbed (i.e., the 2nd root growing period of this study) the biopores are either reused or rebuild at the expense of other pores/biopores. While the large (>0.5 mm Ø) pores are partially destroyed by soil fauna, the proportion of smaller (<0.5 mm Ø) pores increases as a result and due to the action of plant roots, suggesting a highly dynamic equilibrium.

### Cover crop effect

After roots exploring the soil matrix die and decay, they leave behind the biopores of comparable sizes (Lucas, 2022). Indeed, we found differences among the studied plant species in terms of <0.2 mm and 0.2–0.5 mm Ø pores and biopores, the sizes that match well the prevailing root sizes of the investigated cover crops (Figures 1–3). Accordingly, AWP with the largest volume and length of 0.2–0.5 mm Ø roots (Figure 1; Supplementary Figure 4), developed the largest amount of biopores in the 0.2–0.5 mm Ø size class, significantly more than OA, which in generally was characterized by a low root volume.

The direct effect of roots on the pore system, i.e., through the creation of biopores, highly depends on the root growth and volume (Bodner et al., 2014; Bacq-Labreuil et al., 2019; Lu et al., 2020). Our results suggest that, although large root volumes correspond to large biopore volumes in the size classes <0.5 mm Ø, corresponding pore size classes were decoupled from the creation of these biopores and showed even opposite trends. For example, after the first root growth period, the OA soil samples contained the largest pore volumes of <0.5 mm Ø pores, while the corresponding root and biopore volumes were the lowest among the five cover crops observed. In addition, similar to earthworms creating macropores resulting in the loss of smaller pores, roots also compact their surrounding and therefore potentially reduce the volumes of pores of certain size classes (Bruand et al., 1996; Lucas et al., 2019a; Phalempin et al., 2021b; Lucas, 2022). Indeed, roots <0.5 mm in diameter and total root volume and biopore volumes of all size classes correlated negatively with <0.2 mm Ø pores (Figure 4). Therefore the large root volume of DER lead to a high reduction <0.2 mm Ø pores compared to OA, which developed a small root volume in the observed soil depth (Figure 2). This is in line with findings of Bacq-Labreuil et al. (2019) who showed in an experiment with disturbed soil that after 8 weeks of growth Black Oat (*Avena strigosa*) maintained similar macroporosity to that of the non-plant control, while tillage radish (*R. sativus*) reduced macroporosity.

However, roots only create new biopores and potentially compact their surroundings, when they grow into the dense soil matrix (Jin et al., 2017; Lucas, 2022). Indeed, the volume of roots growing in the dense matrix was positively correlated with pores between 0.5–1 mm, while negatively with pores <0.2 mm Ø. The largest number of roots growing into the dense soil were found for AWP (both relatively and in total, Figure 1B; Supplementary Figure S5) and therefore the largest amount of biopores <0.5 mm Ø were created (Figure 3A).

Plants, however, can undergo major morphological changes to adapt to a changing local environment (Burr-Hersey et al., 2017). In Burr-Hersey et al. (2017), the effect on morphological root changes induced by changes in bulk density for three different cover crops showed that tillage radish (*R. sativus*) undergoes greater morphological changes compared to vetch (*V. sativa*), and black oat *(A. strigosa)*, potentially allowing the plant to follow existing macropores. Accordingly, macro- and biopores attract root growth especially under conditions of high soil bulk density and at greater soil depths (White and Kirkegaard, 2010; Han et al., 2015b; Colombi et al., 2017; Atkinson et al., 2020; Zhou et al., 2020). The preferential growth of roots into macropores was visible in the correlation of small root classes (<0.5 mm Ø) with pores of larger sizes, i.e., 0.5–1 mm Ø and 1–2 mm Ø. Indeed, during the 2nd root growing period most roots in our study were found in macropores (~40%) and biopores (~15%, Figure 1B) although these account for, respectively, only approx. 20 and 2% of the total soil volume (Table 1). The reuse of biopores during the 2nd root growing period was comparable to values reported in the literature (White and Kirkegaard, 2010; Kemper et al., 2020), e.g., Kemper et al. (2020) found a reuse between 10 and 22% of biopores by oil radish in the subsoil of a Fluvisol with a silt loam texture. However, how and to what extent roots reuse biopores, heavily depends on plant species (Athmann et al., 2013; Kemper et al., 2020), with taprooted plants seem to reuse biopores more frequently (Kemper et al., 2020). Here we found no significant differences in the share of roots growing into biopores among the studied cover crop species (Figure 1B). This may be explained by the low soil depth of this study thus only minor restrictions to root growth into the soil matrix.

Large volumes of biopores >1 mm Ø, i.e., larger as most roots, may also indirectly result from plant effects on soil fauna. Earthworms may feed on roots and their residues and can prefer crop residues of some plants over others (Curry and Schmidt, 2007). Valckx et al. (2011) revealed that living oat plants were avoided by earthworms. Similar, Euteneuer et al. (2020) showed that radishes were preferred by earthworms compared to oat. These results seem in line with our findings, as in OR a high share of earthworm cast as well as a large amount of biopores >1 mm were found (Figures 3A, 6B), while in OA, especially during the 1st root growing season, only a relatively low amount of biopores was created. Similarly, DER, despite having relatively thick roots, developed a large proportion of biopores >1 mm, which cannot be associated solely with the observed root size classes. The combined effect of roots and soil fauna was presumably the reason for the greatest bioporosity across all cover crop species observed in DER (Table 1).

In summary, soil fauna and roots are linked in multiple ways and the effect of soil fauna and roots cannot be clearly separated. However, the data convincingly demonstrates that cover crop roots create biopores of their size and change the pore system depending on their root characteristics. While DER and OR with their large taproot system created large biopores, the smaller roots of AWP, which preferentially grow into the soil matrix, showed a large effect on biopores between 0.2–0.5 mm Ø, similar to the dense fibrous root system of AR. The largest changes in pore size distribution could be associated with the formation of large biopores, which occurred at the expense of smaller pores. The low volume of the fibrous root system of OA resulted in the smallest bioporosity, preserving small macropores (<0.2 mm Ø). In contrast, large amounts of biopores in all biopore size classes were created in the soil cores of DER, due to both the broad-sized root system and faunal activity. This resulted in the highest reduction in pores <0.2 mm Ø.

## Data availability statement

The raw data supporting the conclusions of this article will be made available by the authors, without undue reservation.

## Author contributions

AK and ML conceptualized the experiment. ML conducted image analysis. ML, AG, and LN did the field sampling. All authors contributed to the article and approved the submitted version.

## Funding

This work is funded in part by USDA-NIFA, Award no. (2018-51106-28779), the USDA-NIFA Program (Award# 2022-67019-36104), the NSF LTER Program (DEB 1027253) at the Kellogg Biological Station, and Michigan State University AgBioResearch.

## Conflict of interest

The authors declare that the research was conducted in the absence of any commercial or financial relationships that could be construed as a potential conflict of interest.

## Publisher’s note

All claims expressed in this article are solely those of the authors and do not necessarily represent those of their affiliated organizations, or those of the publisher, the editors and the reviewers. Any product that may be evaluated in this article, or claim that may be made by its manufacturer, is not guaranteed or endorsed by the publisher.

## Abbreviation

OA: Saber Oat (*Avena sativa*)
DER: Dwarf Essex Rapeseed (*Brassica napus*)
OR: Oilseed Radish (*Raphanus sativus*)
AWP: Austrian Winter Pea (*Pisum sativum*)
