# Supplementary material for: Cover crop influence on pore size distribution and biopore dynamics: Enumerating root and soil faunal effects
Source: Front Plant Sci. 2022 Aug 30;13:928569. doi: 10.3389/fpls.2022.928569 (PMC9491155; doi:10.3389/fpls.2022.928569)
Supplement: Supplementary file 1 [file Presentation_1.pdf]

Supplementary material

Cover crop influence on pore size distribution and biopore dynamics: enumerating root and soil faunal effects

Correspondence:
Maik Lucas
lucasmai@msu.edu

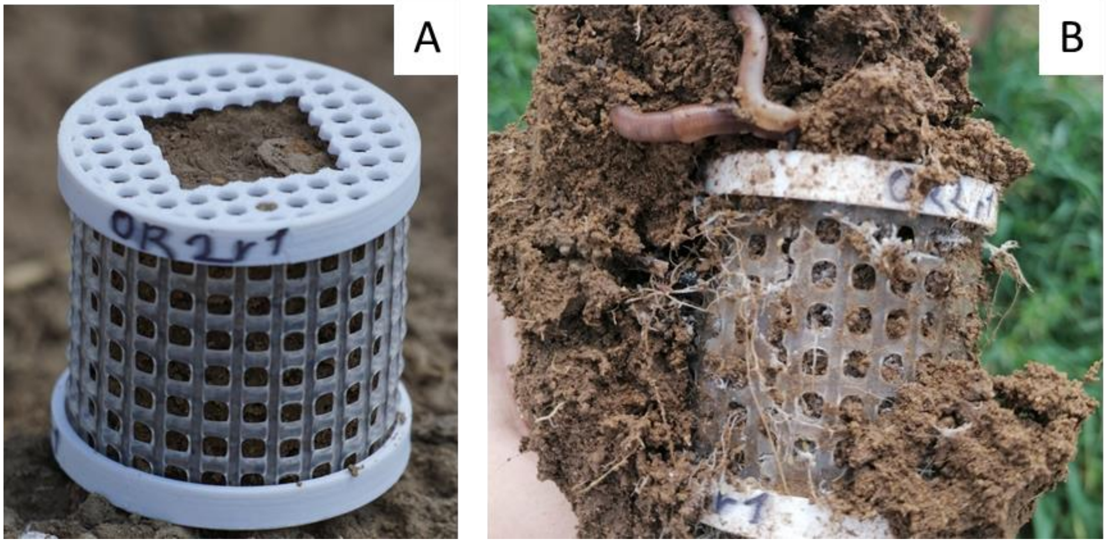

Supplementary Figure 1: Ingrowth cores used for the experiment. A: Before reburied and B after one plant growing period.

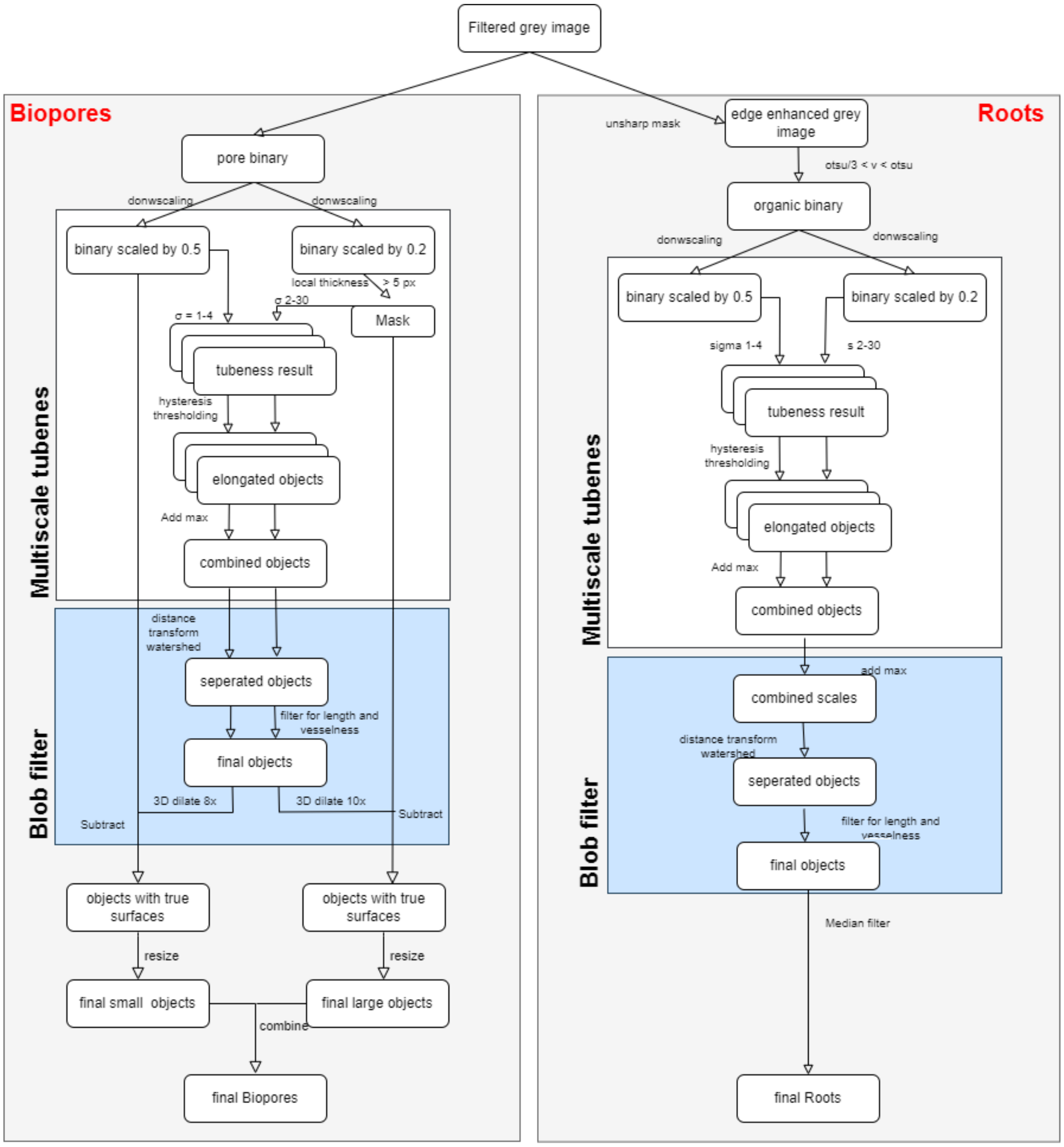

Supplementary Figure 2: Workflow of image segmentation for biopore and roots in FIJI.

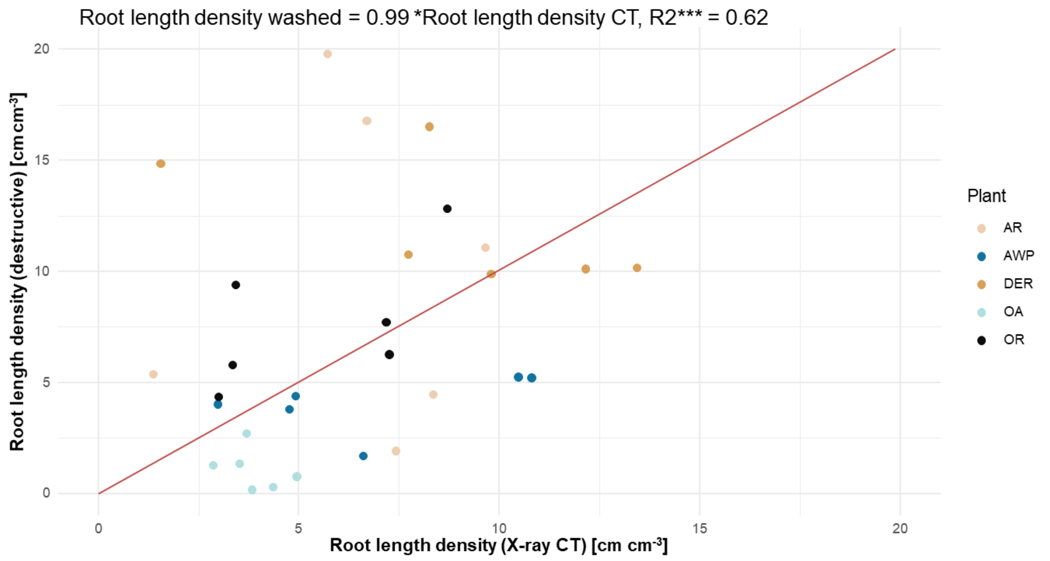

Supplementary Figure 3: Correlation of root length density estimated by CT and root length density estimated destructively.

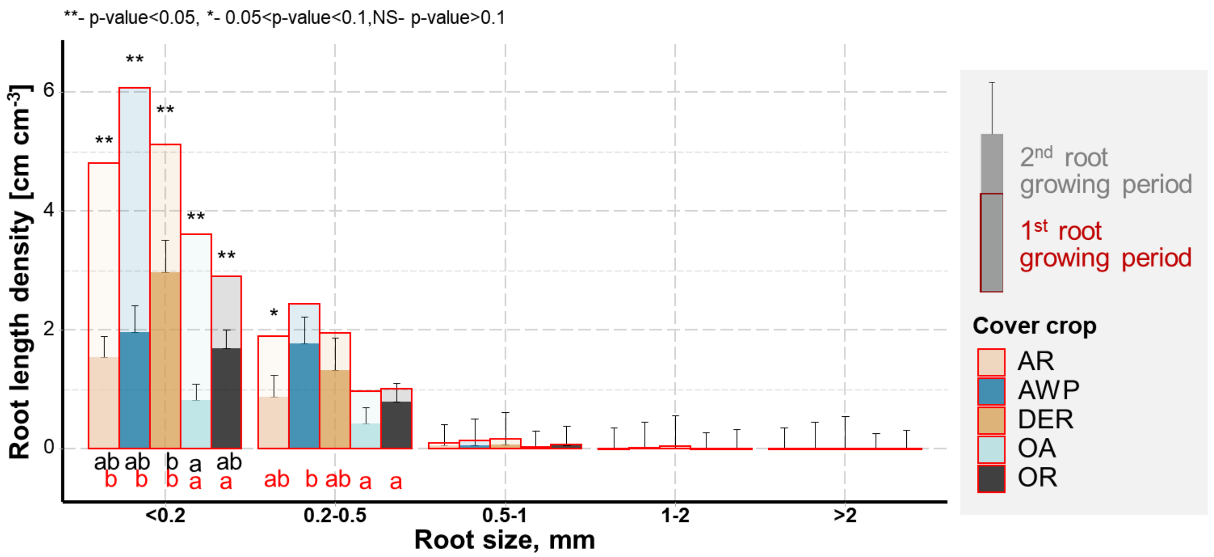

Supplementary Figure 4: Root size distribution (diameter) for root length of the five different cover crops. Letters indicate significant differences within the size classes. Roots from the 1st root growing period are shown in red, new developed roots in in the 2nd root growing period are shown in darker color and corresponding letters in black. Whiskers show standard error of the means in 2021. Stars above the bars show significant differences between the two different root growing periods.

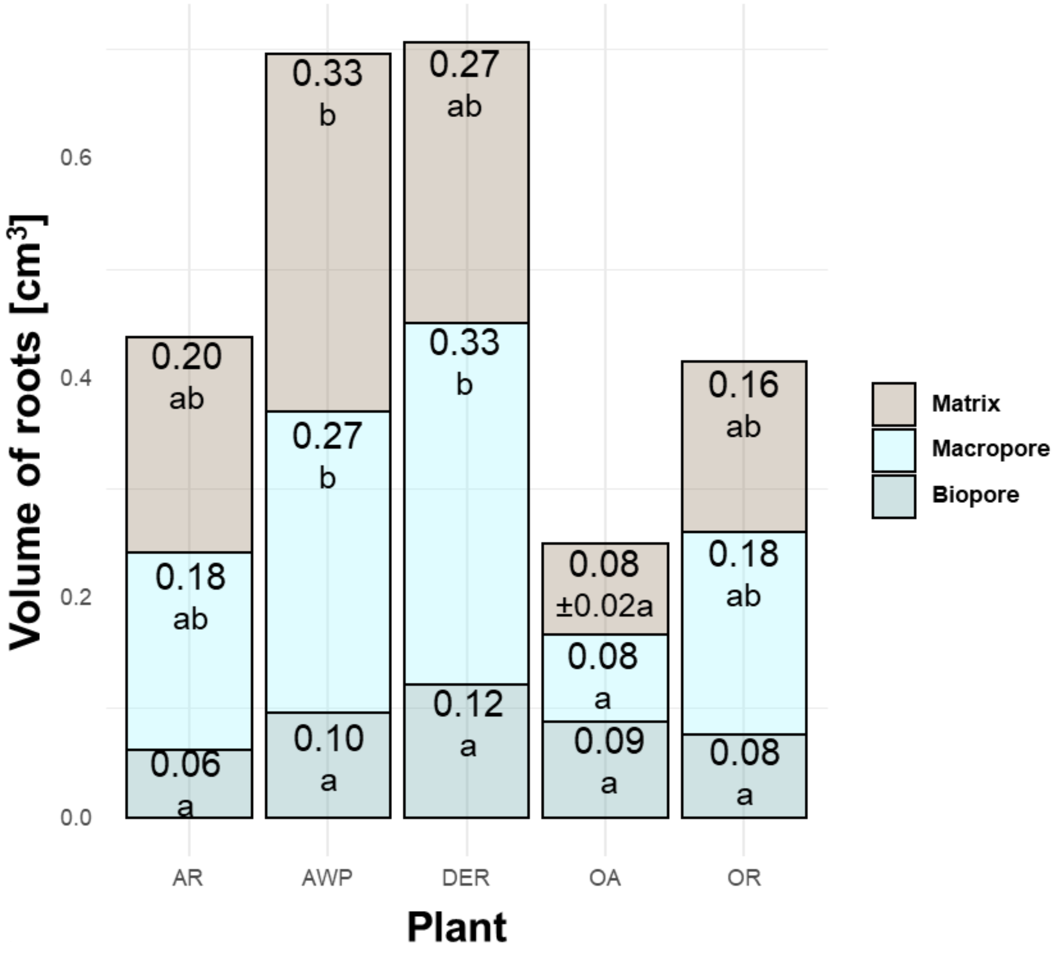

Supplementary Figure 5: Volume of roots growing into different structures of the soil (matrix, pore and biopore) during the 2nd root growing period for the five different cover crops. Different letters indicate significant differences within each structure.
